# Increased BOLD Variability in the Parietal Cortex and Enhanced Parieto-Occipital Connectivity during Tactile Perception in Congenitally Blind Individuals

**DOI:** 10.1155/2012/720278

**Published:** 2012-06-20

**Authors:** Andrea Leo, Giulio Bernardi, Giacomo Handjaras, Daniela Bonino, Emiliano Ricciardi, Pietro Pietrini

**Affiliations:** ^1^Laboratory of Clinical Biochemistry and Molecular Biology, University of Pisa, 56127 Pisa, Italy; ^2^MRI Laboratory, Fondazione Regione Toscana/CNR “G. Monasterio”, 56127 Pisa, Italy

## Abstract

Previous studies in early blind individuals posited a possible role of parieto-occipital connections in conveying nonvisual information to the visual occipital cortex. As a consequence of blindness, parietal areas would thus become able to integrate a greater amount of multimodal information than in sighted individuals. To verify this hypothesis, we compared fMRI-measured BOLD signal temporal variability, an index of efficiency in functional information integration, in congenitally blind and sighted individuals during tactile spatial discrimination and motion perception tasks. In both tasks, the BOLD variability analysis revealed many cortical regions with a significantly greater variability in the blind as compared to sighted individuals, with an overlapping cluster located in the left inferior parietal/anterior intraparietal cortex. A functional connectivity analysis using this region as seed showed stronger correlations in both tasks with occipital areas in the blind as compared to sighted individuals. As BOLD variability reflects neural integration and processing efficiency, these cross-modal plastic changes in the parietal cortex, even if described in a limited sample, reinforce the hypothesis that this region may play an important role in processing nonvisual information in blind subjects and act as a hub in the cortico-cortical pathway from somatosensory cortex to the reorganized occipital areas.

## 1. Introduction

The human cerebral cortex is capable of a high degree of plasticity, a phenomenon based on both functional and structural modifications that allow the brain to adapt to environmental changes as well as to physiological or pathological conditions that may affect the individual [1]. According to this definition, an alteration during brain development may lead to significant changes in brain functional response and network organization as compared to normally developed brains. In this perspective, the study of early sensory deprivation has emerged as an interesting field of research in neuroscience since it represents an exceptional condition to assess; on one side, to what extent the development of the brain functional architecture is independent from that given sensory experience, for example, vision (for a recent critical overview see [2]), on the other side, the potentialities of neural plasticity in reorganizing brain regions primarily affected by sensory deprivation (recently reviewed in [3, 4]). In particular, studies on the congenital lack of vision or its loss at later stages in life have investigated how the absence of vision affects the functional and structural organization of the brain, and which modifications occur in the visual cortical areas as a consequence of the lack of any retinal input [2, 5, 6].

The absence of inputs from the retina since birth induces a cross-modal plastic reorganization in early visual brain areas and a functional rearrangement of their afferent and efferent connections [1, 2, 4, 5, 7, 8]. These primary visual areas are recruited in blind individuals to process stimuli conveyed by nonvisual sensory modalities, that is, the tactile, auditory, and olfactory senses [6, 9–13]. Interestingly, the activation of specific areas in the occipital cortex of blind individuals is not merely an epiphenomenon but rather is fundamental to the new sensory processing, as virtual functional lesions of these areas via transcranial magnetic stimulation (TMS) impair nonvisual performances, such as tactile perception, verb generation, or Braille reading [4, 14–16].

In addition to these cross-modal plastic modifications, the combined study of congenitally blind and sighted individuals also has demonstrated that cortical areas in the ventral and dorsal visual pathways are able to process sensory information regardless of the sensory modality through which such information has been acquired [2, 17]. In fact, while the activation of visual areas during non-visual processing could be ascribed to a visually based imagery in sighted individuals, the observation of an identical response pattern in a group of congenitally blind individuals, who by definition lack vision since birth and therefore do not possess any visually based mental imagery, indicates that these *supramodal* brain regions rely on a more abstract representation of the perceived stimuli, as, for example, in the cases of object category recognition [18, 19], spatial representation [20–24], or motion discrimination [25–27]. While plastic modifications do take place in the blind brain and lead visual areas that are *unimodal* in nature to process stimuli carried by different sensory modalities (*cross-modal plasticity*), at the same time *supramodal* areas develop within visual cortical regions that are ordinarily able to process also non-visual information both in sighted and blind individuals [2]. This more abstract nature of functional cortical organization may enable congenitally blind individuals to acquire knowledge, form mental representations of and interact effectively with an external world that they have never seen. In addition, the concept of *supramodal* organization has been recently extended much beyond the “what” and “where” visual pathways to brain areas associated with other cognitive and affective functions [2].

These observations have raised the question of which neural pathways are responsible for conveying non-visual sensory information to the *supramodal* and to the *cross-modal* specific areas within the visual cortex in congenitally blind individuals [2, 6]. It has been suggested that a rearrangement, or the potentiation, of preexisting cortico-cortical connections, such as in the case of a parieto-occipital pathway for tactile perception, may play a fundamental role in visual cortex recruitment in the blind brain [1, 2, 4, 6]. Consistent with this hypothesis, blind individuals show a specific functional association between parietal and occipital areas as compared to sighted subjects during tactile tasks [16, 28, 29], suggesting that parietal cortex may become a central hub for a more efficient exchange of multimodal information between somatosensory and occipital cortices in the absence of any visual experience.

To test this hypothesis we used a new measure, namely, blood-oxygenation-level-dependent (BOLD) signal variability, that has been recently proposed as an index of brain operative efficiency [30]. Indeed, as shown in some earlier experiments that employed EEG and MEG [31, 32], moment-to-moment variability in brain activity increases with the functional complexity of the cortical networks that subserve a specific function. Thus, local changes in BOLD variability may represent an expression of an increase in the amount of information processed consequent to modifications in the specific functional network configuration [32].

For this reason, we hypothesized that areas within the parietal cortex, because of their enhanced multimodal information processing in congenitally blind as compared to sighted individuals, would show increased BOLD signal variability during tactile processing and a stronger functional correlation with occipital areas, consequent to cross-modal plastic modifications. To this aim, we performed a mean squared successive difference (MSSD) analysis [33, 34] to estimate BOLD temporal variability on data previously collected during a tactile spatial discrimination [20, 24] and a tactile motion perception task [26, 27]. In addition, differences in functional connectivity (FC) of brain areas showing an increased MSSD were also measured in sighted and congenitally blind individuals.

## 2. Methods

### 2.1. Subjects

Seven sighted (2 females,  29 ± 3 yrs) and 4 blind (1 female,  35 ± 15 yrs) right-handed healthy volunteers participated in the tactile spatial discrimination experiment. A partially distinct group of seven sighted (2 females,  27 ± 2 yrs) and four blind (1 female,  37 ± 14 yrs) individuals participated in the tactile motion perception protocol. All blind individuals were blind since birth, except one who became blind within the first two years of life and had no recollection of any visual experience (causes of blindness: congenital glaucoma, retinopathy of prematurity, and congenital optic atrophy). One sighted and two blind volunteers (including the early blind individual) participated in both experiments. All subjects received medical, neurological, and psychiatric examinations and a structural magnetic resonance imaging (MRI) brain scan to exclude any disorder that could affect brain function (other than blindness in the blind group). No subject was taking any psychotropic medication. All subjects gave their written informed consent after the study procedures, and risks involved had been explained (protocol no. 1616/2003 approved by the Ethical Committee of the University of Pisa).

### 2.2. Image Acquisition

We used fMRI to measure brain activity while subjects performed the two experimental paradigms described below. Gradient echo echoplanar images (GRE-EPI-) were acquired with a GE Signa 1.5-T scanner (General Electric, Milwaukee, WI) using the following parameters: repetition time = 3000 ms, 22–26 axial slices, slice thickness = 5 mm, field of view = 24 cm, echo time = 40 ms, flip angle = 90, image plane resolution = 64 × 64 pixels. Voxels were 3.75 × 3.75 × 5 mm. High-resolution T1-weighted spoiled gradient recall images were obtained for each subject to provide detailed brain anatomy.

### 2.3. Tactile Spatial Discrimination

Tactile stimuli were wooden squares and cubes with three or five Velcro-covered target-squares/cubes [24]. Each matrix was randomly presented to the subjects by using a wooden pole with a Plexiglas platform on one end upon which the wooden matrix was attached with Velcro. In this study, we used two-dimensional (2D) 5 × 5 matrices and three-dimensional (3D) 3 × 3 × 3 matrices, comparable in terms of number of targets and number of potential combinations of target location. In order to control for any sequence effects, the presentation of different matrices (2D versus 3D) and number of targets (3 versus 5), as well as the foot code response, were randomized within and between subjects. Each stimulus was created in order to be different from the others, even if translated or rotated. Matrices were presented for 10 s during the tactile task, with an interstimulus interval of 5 s. Each time series consisted of 16 consecutive matrices with three/five targets in counterbalanced order. Subjects were instructed to explore the matrices with both hands, to generate a mental image of the perceived stimulus and to maintain it in memory for a comparison with the next stimulus. During baseline and interstimuli periods, subjects were asked to rest their arms along their side and keep them still. Sighted subjects were required to keep their eyes closed while performing the task. During the one-back tactile recognition task, volunteers tactilely explored 16 sequential matrices in 6 to 8 experimental runs and indicated whether the last matrix was the same or different as compared to the previous one, by pressing foot pedals with the right (e.g., same) or left (e.g., different) foot.

### 2.4. Tactile Motion Perception

Tactile stimuli were moving or static Braille-like dot patterns (diameter = 1 mm, height = 1 mm) randomly displaced on a plastic flat surface. The average distance was 9 mm so the stimuli could not recall any letter of the Braille alphabet in blind subjects. Two types of motion were used during the task: horizontal translation (left to right and right to left at about 2.2 cm/s) and rotation (clockwise and counterclockwise at about 93.5°/s). Tactile stimuli were randomly presented using an MRI compatible device on a polystyrene table placed over the subjects' legs. Participants' hands lay on the table with the index and middle fingers touching the plastic surface with dot patterns. Moving stimuli were presented in 8 to 40 s blocks separated by intervals with static stimuli of varying duration (11 ± 10 s). Type of movement, direction of movement, and side of stimulation (right hand or left hand) were randomized and counterbalanced within and across subjects. Each time series began and ended with 30 s of static stimuli [26, 27].

### 2.5. Data Preprocessing

We used AFNI and SUMA software packages to preprocess, analyze, and view functional imaging data (http://afni.nimh.nih.gov/afni/  [35]). Acquired raw data were reconstructed, coregistered to the volume collected nearest in time to the high-resolution anatomy, phase-shifted using Fourier transformation to correct for slice acquisition time, spatially smoothed (FWHM = 8 mm), and normalized to estimate the percent signal change at each time point. Individual preprocessed data were then transformed into the Talairach and Tournoux Atlas [36] coordinate system and resampled into 3 mm^3^ voxels. Finally, voxel time series were further adjusted by regressing out motion correction parameters, a polynomial function modeling the BOLD drifting effect and white matter (WM) and cerebrospinal fluid (CSF) time series [30]. For each experiment WM and CSF time courses were extracted from two single voxels, respectively, located in the corpus callosum and the ventricles of a common template obtained by merging spatially normalized anatomical images from all the participants. Finally, a low pass filter was applied. Results obtained in subsequent analyses were anatomically localized on the group-averaged Talairach-transformed T1-weighted images.

### 2.6. BOLD Temporal Variability Analysis

We used the mean squared successive difference (MSSD) measure to calculate BOLD signal temporal variability in every subject and for each experimental condition. For each individual run, MSSD was computed over the entire preprocessed activation time course using a custom-built function in MATLAB (The MathWorks, Inc.).

For each subject, MSSD values were averaged across different runs of the same experimental protocol, and nonparametric Mann-Whitney tests were used to look for any potential difference between blind and sighted subjects. Significance threshold was set at corrected *P* < 0.05, calculated with a Monte-Carlo simulation run via *3dClustSim* program in AFNI, with a voxelwise threshold of *P* < 0.02, which resulted in a minimum cluster volume of *k* < 111 voxels.

Thresholded sighted versus blind MSSD comparison maps of the two different experiments were used to compute a conjunction map (logical AND) to identify regions that showed similar differences in signal variability across conditions (small volume correction *P* value < 0.001, minimum cluster volume *k* > 28 voxels, as calculated on the sighted versus blind MSSD comparison map of the spatial discrimination condition).

### 2.7. Functional Connectivity Analysis

Cortical areas whose BOLD signal variability was significantly different between sighted and blind individuals during both the tactile spatial discrimination and the tactile motion perception experiments were used as seed regions of interest (ROI) for a functional connectivity analysis. Specifically, for each subject and condition the Pearson's correlation coefficient was computed between the BOLD signal time course (obtained concatenating all task-related functional runs) of the ROI and the time course of all the other voxels of the brain.

To identify the significant pattern of functional connectivity for each group and each experimental condition, we converted correlation coefficients of each subject into *Z* scores using Fisher's *Z* transformation and then performed one-sample group *t*-tests. Significance threshold was set at corrected *P* < 0.05, obtained using a voxelwise threshold of *P* < 0.01 and a cluster greater than 66 voxels. Furthermore, in order to determine significant differences in functional connectivity between congenitally blind and sighted individuals in each condition, unpaired nonparametric Mann-Whitney tests were performed to compare the two groups during both tactile experiments (voxel-wise *P* < 0.05, *k* > 30 voxels).

## 3. Results

### 3.1. BOLD Signal Variability in Congenitally Blind and Sighted Individuals

**Figure 1:**
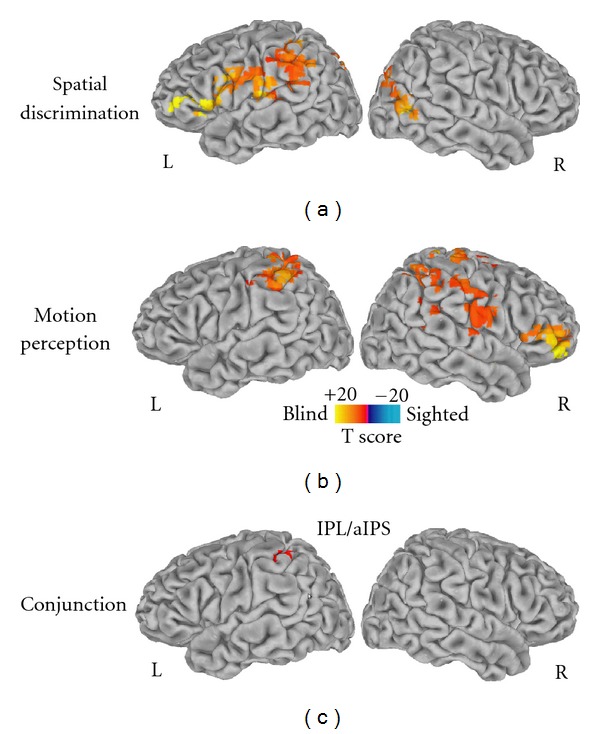
Mean squared successive difference (MSSD) significant (corrected *P* < 0.05) differences between blind and sighted individuals during (a) the tactile spatial discrimination and (b) tactile motion perception experiments. (c) The conjunction map (logical AND) obtained from the thresholded sighted-versus-blind MSSD maps revealed a region located in left inferior parietal and anterior intraparietal cortex (IPL/aIPS) that showed similar differences in signal variability in the tactile spatial and motion discrimination experiments (*k* > 30 voxels). Spatially normalized MSSD differences are projected onto single-subject left and right hemisphere templates in the Talairach space.

During both experiments the blind subjects showed a significantly higher MSSD measure in a number of cortical brain areas as compared to the sighted individuals. Specifically, during the tactile spatial discrimination task, blind individuals had a significantly greater (corrected *P* < 0.05) signal variability in the left superior and bilateral inferior parietal, left superior frontal, right middle temporal, bilateral superior temporal, lingual, medial frontal and cingulate cortex, precuneus and cuneus (Figure 1(a)). During the tactile motion perception task, blind individuals showed a significantly greater (corrected *P* < 0.05) signal variability in the bilateral superior and inferior parietal cortex, left lingual, right superior temporal, postcentral, inferior frontal, and anterior cingulate cortex, as compared to the sighted group (Figure 1(b)).

The conjunction map computed from the sighted-versus-blind MSSD contrast maps obtained for the two tactile experimental protocols revealed a common cluster (small volume correction, *P* < 0.02; Figure 1(c)) of greater BOLD variability in the blind as compared to sighted individuals located in left inferior parietal and anterior intraparietal cortex (Talairach coordinates of the center of mass were *x* = −40, *y* = −43, *z* = 49).

### 3.2. Functional Correlation between the Left Parietal Cortex and Occipital Areas in the Congenitally Blind and Sighted Individuals

**Figure 2:**
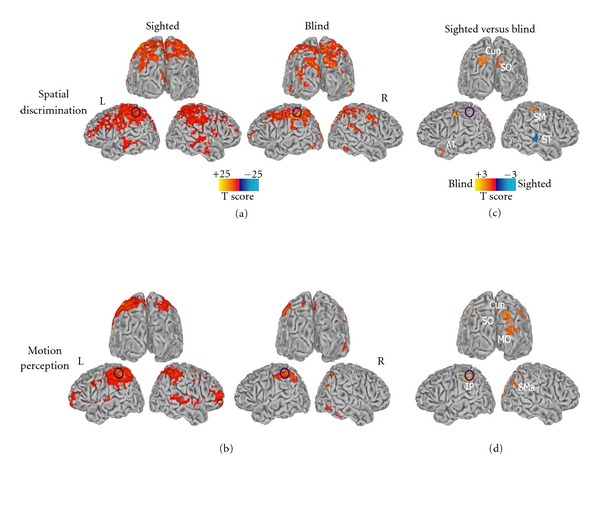
Group functional connectivity (FC) maps obtained for sighted (first column) and blind (second column) individuals during (a) the tactile spatial discrimination and (b) the tactile motion perception tasks (corrected *P* < 0.05). The third column (c)-(d) shows the differences in functional connectivity between the two groups (uncorrected *P* < 0.05, *k* > 30 voxels). The left inferior parietal seed ROI has been indicated with a purple circle. Spatially normalized maps are projected onto single-subject left and right hemisphere templates in the Talairach space in frontal and lateral views. Cun: cuneus; SO: superior occipital; MO: middle occipital; SMa: supramarginal; IP: inferior parietal; ST: superior temporal; AT: anterior temporal.

We performed a functional connectivity analysis using the left inferior parietal and anterior intraparietal cluster as a seed ROI for each group and each experimental condition. In both the blind and sighted individuals, during the tactile spatial (Figure 2(a)) and tactile motion (Figure 2(b)) discrimination tasks, the parietal ROI was significantly correlated (corrected *P* < 0.05) with a number of brain areas including inferior/superior parietal, middle/superior temporal, middle/superior and anterior frontal, motor and cingulate cortex, and clusters in the occipital regions. Interestingly, blind subjects showed a greater correlation between the left inferior parietal lobule and visual areas, comprising bilateral cuneus/superior occipital (Talairach coordinates of the peak voxels: *x* = −16, *y* = −82, *z* = 32; *x* = 7, *y* = −85, *z* = 41) during the spatial discrimination task and bilateral cuneus, superior and middle occipital (Talairach coordinates of the peak voxels: *x* = 23, *y* = −82, *z* = 26; *x* = −25; *y* = −70, *z* = 38), precuneus (Talairach coordinates *x* = −5, *y* = −56, *z* = 29), and right supramarginal (Talairach coordinates *x* = 44, *y* = −55, *z* = 29) cortex during the motion perception task (Figures 2(c)–2(d)). Additional regions in bilateral sensorimotor and left anterior temporal cortex (spatial discrimination) and in bilateral inferior parietal cortex and anterior cingulate (motion perception) showed a greater correlation with the seed ROI in blind individuals, while in bilateral medial prefrontal and right superior temporal cortex during the spatial discrimination task in sighted individuals (Figures 2(c)-2(d)).

## 4. Discussion

The aim of the present study was to examine whether the lack of visual experience since birth may lead to changes in neural efficiency, as measured by the BOLD signal temporal variability, within the parietal cortex and in its connections with occipital cortical areas in relation to the processing of non-visual sensory information. As a matter of fact, despite the growing number of studies indicating the existence also in blind individuals of *supramodal* areas within the visual cortex capable of processing information conveyed by different sensory modalities [2] and the potential additional development of *cross-modally*-reorganized occipital clusters [3, 5, 21], the specific pathway(s) that carry such non-visual sensory information to the occipital cortex are still a matter of wide debate. Indeed, direct connections between different primary sensory areas, subcortico-cortical loops, or cortico-cortical pathway(s) are all considered potential mechanisms to explain how non-visual sensory inputs reach the “visual” cortex [3, 6].

As far as tactile processing in the occipital areas is concerned, the hypothesis of a cortico-cortical connection has been supported by the results of experiments that used TMS to induce temporary functional lesions during Braille reading [4, 15, 37]. In congenitally blind individuals, parietal activation in response to tactile letter detection precedes occipital activation associated with letter identification, thus suggesting that tactile information reaches the occipital cortex through a parieto-occipital pathway [1]. Additionally, other tactile experimental paradigms in early blind participants confirmed a reinforced functional coupling between occipital and parietal cortical areas, without any subcortical involvement [4, 15, 27–29]. For instance, a connectivity analysis during electrotactile stimulation of the tongue showed that anterior areas of the parietal cortex had an increased correlation in activity with posterior parietal cortex and the visual occipital cortex of trained blind individuals [28]. Consistently, in a distinct tactile motion perception experiment in which subjects had to discriminate motion of plastic dots under their fingertips [25], we found that somatosensory areas showed extensive bilateral connections with contiguous posterior parietal and intraparietal regions, and with middle temporal and lateral occipital areas in both sighted and congenitally blind individuals, supporting a cortico-cortical pathway from primary somatosensory cortex through parietal regions to occipital areas [27].

On the other hand, we cannot exclude that other sensory modalities, such as auditory inputs, may follow distinct pathways of sensory integration. Indeed, the spatial processing of sounds in early blind and sighted individuals was impaired only when TMS was applied at short latencies to the right dorsal occipital cortex, but not when it was applied to the right intraparietal region [22, 38], thus suggesting that sounds may reach occipital regions in the blind brain either via subcortical connections or direct projections from the auditory cortex.

In light of these preliminary observations on the occipital processing of somatosensory information, we hypothesized that, as a consequence of plastic rearrangements, parietal areas in congenitally blind individuals would elaborate a greater amount of information during tactile tasks and would be characterized by specific functional networks with strengthened occipitoparietal connectivity. Parietal areas, including the superior parietal and the intraparietal cortex, have been proposed as hubs of integration for multimodal inputs, and many studies have found connections of these subregions with primary areas of different sensory modalities both in monkeys and in humans (reviewed in [39, 40]). Therefore, parietal regions multisensory in nature which are expected to receive and integrate different inputs may show a more efficient processing and a greater capacity to switch between different network configurations. To test this hypothesis, in the present study we compared BOLD signal temporal variability, a recently proposed index of functional brain efficiency [32, 41], across sighted and congenitally blind individuals during two different tactile tasks: spatial discrimination and motion perception.

Variability in BOLD signal is thought to increase with the functional complexity of the cortical networks that subserve a specific function, as shown in some earlier experiments which employed EEG and MEG [31, 32, 42], though the physiological meaning of this measure still remains to be fully understood [42]. For instance, by studying populations at different ages, Garrett and colleagues recently showed that the noise (i.e., temporal variability) in BOLD signal is greater in young highly performing adults as compared to older adults [30, 32]. Moreover, a higher variability in certain brain areas was associated with superior behavioral performances, suggesting a correlation between this parameter and operative brain functional efficiency [41]. In the present study, BOLD signal variability was assessed by computing the mean of the square differences between the values of BOLD signal at two successive time points (i.e., MSSD) [33, 34].

Our results showed that during both the tactile spatial discrimination and motion perception conditions, the blind individuals were characterized by increased signal variability in brain areas distributed over prefrontal, parietal, occipital and temporal cortex. Differences in MSSD scores between the two groups were more evident in the tactile spatial discrimination task than in the motion perception condition, mainly in temporal, prefrontal and occipitoparietal areas. In order to avoid specific effects dependent on the distinct experimental conditions, we performed a conjunction analysis to identify common areas of increased MSSD in the congenitally blind group. A significant cluster of overlapping in group MSSD differences was localized in the left inferior parietal and anterior intraparietal cortex.

To verify whether such differences in BOLD signal variability were also associated with changes in the functional network organization, a functional connectivity analysis using the identified parietal cluster as a seed region of interest also was carried out. Results showed a more extended inclusion of occipital regions in the connectivity network of this parietal region in the blind as compared to sighted individuals. In general, congenitally blind individuals demonstrated enhanced connectivity between brain areas processing different sensory inputs as compared to sighted [43, 44] or novel pattern of correlations between cross-modal reorganized occipital areas and cortical areas conveying or elaborating non-visual stimuli [27].

Taken together, our MSSD and functional connectivity results suggest a reinforced integrative role of the parietal cortex during tactile perception in congenitally blind individuals as compared to the sighted ones. Moreover, our results provide novel support to the potential role of the parietal cortex in conveying tactile and maybe other non-visual information to striate and extrastriate occipital regions in blind individuals. In fact, although a concomitant involvement of a subcortical loop between the somatosensory and occipital areas may exist [2, 45], these results suggest that important functional plastic modifications occur in the parietal cortex in congenitally blind individuals, including changes in the functional connectivity network, which is rewired to reach occipital areas.

Notably, it should be emphasized that the measures of BOLD temporal variability are independent of task-related BOLD responses and appear more to provide information about the level of functional integration other than the recruitment of a specific brain region during distinct experimental tasks [30, 41, 42]. Therefore, these measures do not directly reflect task-related perceptual and cognitive processing. Furthermore, the original studies on BOLD signal variability evaluated the effects of different cognitive tasks on this measure [30, 41, 42]. The authors showed a substantial independence of the age-related differences in BOLD variability from the specific task employed. Here, to properly avoid any specific effects dependent on the two experimental conditions, we also performed a conjunction analysis to identify common areas of increased MSSD in the congenitally blind groups.

Sample sizes may represent a limitation of our analysis, as four blind individuals per experimental protocol is a relatively small number of subject are for an fMRI study nowadays, and the results reported here would need to be replicated in a larger cohort. Nonetheless, congenitally blind individuals represent an exceptionally rare population that should comply with strict selection criteria from a medical (e.g., no other neurological disorders, drug-free) and demographic (e.g., independent living conditions, socially integrated) perspective. Even if limited samples could be sufficient to respond to specific hypothesis-driven protocols (e.g., [18]), some methodological considerations have been however taken into account, such as the use of nonparametric comparisons.

## 5. Conclusions

In summary, these findings, though acquired in a relatively limited sample and therefore in need of replication in larger groups, expand the current knowledge on the functional reorganization that occurs in the brain of congenitally blind individuals, as they demonstrate that during both tactile spatial discrimination and motion perception tasks, the inferior parietal and anterior intraparietal cortcies were characterized by increased BOLD signal temporal variability and relevant functional network modifications in the congenitally blind as compared to the sighted subjects. Overall, these findings support a role for parietal cortex in conveying nonvisual-information to visual cortex after cross-modal plastic modifications in the brain of visually deprived individuals.
